# Association of Metformin Use with Outcomes in Advanced Endometrial Cancer Treated with Chemotherapy

**DOI:** 10.1371/journal.pone.0147145

**Published:** 2016-01-20

**Authors:** Obiageli Ezewuiro, Tatyana A. Grushko, Masha Kocherginsky, Mohammed Habis, Jean A. Hurteau, Kathryn A. Mills, Jessica Hunn, Olufunmilayo I. Olopade, Gini F. Fleming, Iris L. Romero

**Affiliations:** 1 Department of Medicine, University of Chicago, Chicago, Illinois, United States of America; 2 Department of Public Health Sciences, University of Chicago, Chicago, Illinois, United States of America; 3 Department of Obstetrics and Gynecology, Gordon Center for Integrative Science, University of Chicago, Chicago, Illinois, United States of America; 4 Department of Obstetrics and Gynecology, NorthShore University Health System, Chicago, Illinois, United States of America; Zhejiang University School of Medicine, CHINA

## Abstract

There is increasing evidence that metformin, a commonly used treatment for diabetes, might have the potential to be repurposed as an economical and safe cancer therapeutic. The aim of this study was to determine whether stage III-IV or recurrent endometrial cancer patients who are using metformin during treatment with chemotherapy have improved survival. To test this we analyzed a retrospective cohort of subjects at two independent institutions who received chemotherapy for stage III-IV or recurrent endometrial cancer from 1992 to 2011. Diagnosis of diabetes, metformin use, demographics, endometrial cancer clinico-pathologic parameters, and survival duration were abstracted. The primary outcome was overall survival. The final cohort included 349 patients, 31 (8.9%) had diabetes and used metformin, 28 (8.0%) had diabetes but did not use metformin, and 291 (83.4%) did not have diabetes. The results demonstrate that the median overall survival was 45.6 months for patients with diabetes who used metformin compared to 12.5 months for patients with diabetes who did not use metformin and 28.5 months for patients without diabetes (log-rank test comparing the three groups *P* = 0.006). In a model adjusted for confounders, the difference in survival between the three groups remained statistically significant (*P* = 0.023). The improvement in survival among metformin users was not explained by better baseline health status or more aggressive use of chemotherapy. Overall, the findings in this retrospective cohort of endometrial cancer patients with stage III-IV or recurrent disease treated with chemotherapy indicate that patients with diabetes who were concurrently treated with metformin survived longer than patients with diabetes who did not use metformin.

## Introduction

Metformin (N, N dimethybiguanide) is an oral antidiabetic drug in the biguanide class. The compound was clinically developed as a diabetes treatment in the 1950s by Jean Sterne, who named it Glucophage, meaning glucose eater [1]. Metformin was approved by the Food and Drug Administration (FDA) for diabetes treatment in 1995, and was later recommended as first line therapy for type II diabetes by the American Diabetes Association [2]. In addition, metformin is commonly used to treat polycystic ovary syndrome, a use not approved by the FDA [3].

A protective effect of metformin against cancer was first suggested in 2005 in a case-control study by Evans et al., who reported that patients with type II diabetes treated with metformin had a reduced risk of cancer [4]. Several reports followed with similar findings [5–7]. In 2013, a meta-analysis of six observational studies (24,410 patients) found that use of metformin was associated with reduced risk of death due to cancer (OR 0.65, 95% CI 0.53–0.80; P < .0001) [8]. It is hypothesized that metformin’s anti-cancer effects are mediated by systemic effects via decreasing both insulin and glucose and by direct effects on cancer cells through activation of critical signaling pathways, including AMP-activated kinase (AMPK) [9].

As recently reviewed [10,11], several *in vitro* and observational studies have evaluated the potential benefits of metformin in the prevention or treatment of gynecologic cancers. However, the biologic rationale for using metformin in cancer may be strongest for endometrial cancer. Endometrial cancer risk is increased in the setting of obesity [12] and hyperinsulinemia [13], both risk factors could be modified by metformin, since it is used for diabetes and metabolic syndrome treatment. In studies using endometrial cancer cell lines, investigators have reported a dose-dependent inhibition of cell growth with metformin treatment [14–18]. In mouse models, metformin treatment reduced endometrial tumor weight by nearly 50% in a xenograft model [17], but did not improve response to carboplatin in a study using two patient-derived xenograft mouse models [19]. Two retrospective analyses of patient cohorts have evaluated the association between metformin use and endometrial cancer survival. One study, which primarily included women with stage I disease (79%), reported that metformin use was associated with improved recurrence-free survival and overall survival [20]. A second study, which also primarily included women with early-stage disease (70% stage I disease), found improved overall survival (recurrence was not assessed), but only among metformin users with non-endometrioid histology [21]. These studies did not include a sufficient number of women with stage III-IV or recurrent disease to allow an analysis of the effect of metformin in this population.

If metformin proves to be a viable anti-cancer therapeutic, its greatest impact in endometrial cancer might be in the setting of stage III-IV or recurrent disease, since this subset of patients has a five-year survival rate of only 17%, as compared to a 95% survival rate for early stage disease [22]. Currently, there is little, if any, data on whether metformin use by endometrial cancer patients with stage III-IV or recurrent disease might improve survival. To gather information on this question, we analyzed the effects of metformin use in a retrospective cohort of endometrial cancer patients with stage III-IV or recurrent disease. Since one hypothesis is that metformin might act as a chemosensitizer, we limited this analysis to patients treated with chemotherapy. Using this endometrial cancer patient cohort, we then tested if metformin use was associated with improved overall survival.

## Materials and Methods

### Study Cohort

This is a retrospective cohort study of patients treated by gynecologic and medical oncologists for endometrial cancer at The University of Chicago Medical Center (UCMC) from 1992 to 2013 and NorthShore University Health System (NSUHS) from 2000 to 2013. The Institutional Review Boards at University of Chicago Medical Center (#IRB12-1157) and NorthShore University Health System (#EH14-374) approved the study and both Institutional Review Boards waived the need for written informed consent. The study followed the STROBE (STrengthening the Reporting of OBservational studies in Epidemiology) guidelines. To identify subjects, the cancer registries at the two independent hospitals were searched using the terms: endometrial cancer, corpus uteri, stage III, stage IV, and recurrence. Subjects were included in the study if they were diagnosed with the 2009 International Federation of Gynecology and Obstetrics (FIGO) stage III, IV or recurrent disease and were treated with chemotherapy for this diagnosis. Carcinosarcomas were included, but pure sarcomas were not. Subjects were excluded if they had previously received chemotherapy for early-stage disease. For each patient, demographic, endometrial cancer clinico-pathologic parameters, treatment, and outcomes were collected and managed using Research Electronic Data Capture (REDCap) hosted at University of Chicago [23]. Data entry was performed by one group of investigators (O.E., M.H., K.A.M., and J.H.) and was reviewed and verified for accuracy by other investigators (T.A.G., G.F.F. and I.L.R).

### Variables

A review of the medical record was completed for each subject identified in the query of the cancer registries. For all patients included in the study, pathology reports were evaluated to confirm an endometrial cancer diagnosis. Demographic information, start date for chemotherapy, type of chemotherapy, date of last follow-up, and date of death were abstracted from medical records or the cancer registries. Diabetes treatment, medical comorbidities, and body mass index (BMI) was also abstracted. Data was entered directly into REDCap. If data was missing, another investigator performed a second chart review. Inpatient and outpatient medical records were reviewed. In consideration of immortal time bias [24], we determined whether the diabetic treatment at the time of the diagnosis of stage III-IV or recurrent endometrial cancer was the same as the diabetic treatment at the time of death or the end of the study. A minority of subjects changed diabetes treatment during the follow-up period, however, we could not determine when during the course of follow-up the change occurred.

### Statistical Analysis

The patient’s information was de-identified prior to analysis. For statistical analysis, patients were classified into three groups: patients with diabetes who were using metformin (metformin group), patients with diabetes who were not using metformin (non-metformin group), and patients without diabetes (no diabetes group). Demographic and clinico-pathologic variables were compared among the groups, using analysis of variance (ANOVA), Kruskal-Wallis, and Fisher’s exact tests. The primary outcome measure was overall survival (OS). OS was calculated from the date of the start of chemotherapy for stage III-IV disease until the date of death from any cause. Survival times were censored at the date of last follow up if the patient was still alive. OS was estimated and plotted using the Kaplan-Meier method, and compared between groups with log-rank tests. Cox proportional hazards models were used to estimate hazard ratios (HRs) and their 95% confidence intervals (CI's) for OS. Multivariate models were also fitted and adjusted for other prognostic factors: study site, BMI, race, age, and FIGO stage. The race variable was self-reported by patients and dichotomized as White vs. Black and Other, due to the small sample size of the "Other" race group and because its OS was similar to the Black group. Model selection was performed using backward elimination. Statistical significance was defined as *P*<0.05. All analyses were performed using Stata version 13 (StataCorp, College Station, TX).

## Results

### Patient Demographics and Clinical Characteristics

**Fig 1:**
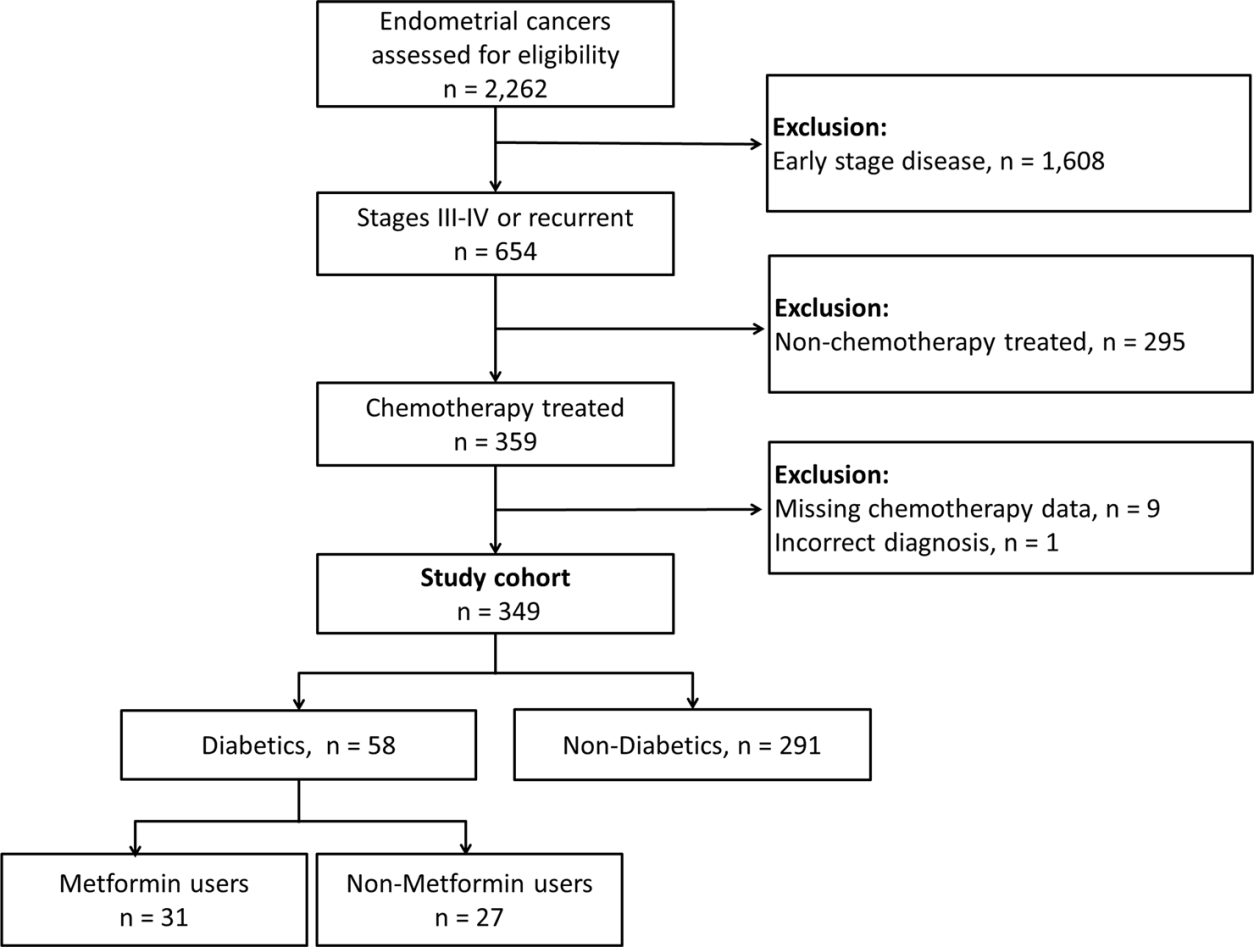
Study selection criteria and primary exposures.

From tumor registries at the two institutions, 2,262 patients (1,310 at UCMC and 952 at NSUHS) treated for endometrial cancer during the study period were identified (Fig 1). Of these, 1,608 subjects had stage I or II disease and were excluded. From the remaining 654 patients, an additional 395 were excluded for: not having received chemotherapy (n = 295), missing dates of chemotherapy (n = 9), or incorrect diagnosis (n = 1). The final cohort included 349 subjects with stage III-IV or recurrent endometrial cancers that met study inclusion criteria.

At the two sites, 40.1% (UCMC) and 6.5% (NSUHS) of the patients were Black (*P* < .001). At UCMC, 85.0% had stage III-IV disease and 15.0% had recurrent disease, whereas at NSUHS, 92.7% had stage III-IV disease and 7.3% had recurrent disease (*P* = 0.041). The two sites were similar with respect to age at diagnosis, diabetes and metformin use, smoking status and tumor histology. Median follow-up for the 108 patients who were still alive at the end of the study (53 at UCMC and 55 at NSUHS) was 37.0 months (range 0.6 to 168.7). Follow-up was longer at UCMC (43.0 months vs. 31.6 months; *P* = 0.02).

Of the 349 patients with stage III-IV or recurrent endometrial cancer included in the analysis, 31 (8.9%) had diabetes and used metformin, 27 (7.7%) had diabetes and did not use metformin (referred to as “non-metformin” group), and 291 (83.4%) did not have diabetes (Table 1). The three groups were similar with respect to age at diagnosis, tobacco use, histologic subtype, use of platinum-based chemotherapy, and stage III-IV or recurrent disease at time of chemotherapy. The median follow-up was similar among the three groups (metformin: 50 months, non-metformin: 54 months, no diabetes: 33 months; *P* = 0.10). There was a significant difference among the racial categories, with a higher portion of Black patients in the non-metformin group than in the metformin group (*P* < .001). Subjects with diabetes also had significantly higher BMI than subjects without diabetes (P = 0.023).

**Table 1:** Patient demographics and baseline characteristics of study cohort.

		Metformin Group	Non-metformin Group	No Diabetes Group	Total	*P*-value
**Cases**		31 (8.9)	27 (7.7)	291 (83.4)	349	
**Age at chemo (years)**		65±11	65±11	63±11	64±11	.77
**BMI**		35.3±9.7	33.3±8.1	30.8±9.1	31.4+9.2	.023
**Obesity**						.020
	BMI < 30	9 (29)	9 (33)	143 (49)	161 (46)	
	BMI ≥ 30	21 (68)	15 (56)	114 (39)	150 (43)	
	Unknown	1 (3)	3 (11)	34 (12)	38 (11)	
**Race**						< .001
	White	21 (68)	7 (26)	210 (72)	238 (68)	
	Black	6 (19)	19 (70)	73 (25)	98 (28)	
	Other	2 (6)	1 (4)	4 (1)	7 (2)	
	Unknown	2 (6)	0	4 (1)	6 (2)	
**Ethnicity**						.720
	Non-Hispanic	30 (97)	27 (100)	285 (98)	342 (98)	
	Hispanic	1 (3)	0	6 (2)	7 (2)	
**Tobacco Use**						.203
	Never used	14 (45)	12 (44)	128 (44)	154 (44)	
	Current user	1 (3)	4 (15)	21 (7)	26 (7)	
	Previous user	13 (42)	5 (19)	74 (25)	92 (26)	
	Unknown	3 (10)	6 (22)	68 (23)	77 (22)	
**Histology**						.616
	Endometrioid	10 (32)	6 (22)	88 (30)	104 (30)	
	Serous	6 (19)	10 (37)	80 (27)	96 (28)	
	Clear cell	3 (10)	1 (4)	13 (4)	17 (5)	
	Carcinosarcoma	2 (6)	4 (15)	42 (14)	48 (14)	
	Adenocarcinoma, NOS	4 (13)	4 (15)	28 (10)	36 (10)	
	Other	6 (19)	2 (7)	40 (14)	48 (14)	
**Received platinum-based chemotherapy**						.190
	Yes	23 (74)	19 (70)	243 (84)	285 (82)	
	No	7 (23)	6 (22)	38 (13)	51 (15)	
	Unknown	1 (3)	2 (7)	10 (3)	13 (3)	
**Stage/Recurrence**						.190
	III	15 (48)	13 (48)	145 (50)	173 (50)	
	IV	11 (35)	7 (26)	115 (40)	133 (38)	
	Recurrent	5 (16)	7 (26)	31 (11)	43 (12)	

Data are n (%) or mean ± standard deviation unless otherwise specified

* *P*-values are from Fisher’s exact or Kruskal-Wallis test, excluding missing values

NOS: not otherwise specified

Total percent may not add up to 100 due to rounding

The baseline health of the metformin and non-metformin users was comparable as reflected by similar medical comorbidities (S1 Table). Seventy-eight percent of subjects stayed on the same diabetic treatment from the time chemotherapy started until the time of last follow-up. The diabetic treatments used in the non-metformin group included: sulfonylureas (n = 10), insulin (n = 7), diet control (n = 8), and thiazolidinedione (n = 3), 6 of these subjects changed diabetes treatment before the time of last follow-up, but none changed to metformin. Among the 6 metformin users that changed medications, 2 discontinued metformin and changed to diet control, 2 changed to sulfonylureas, and 2 changed to insulin.

### Survival Analysis

**Fig 2:**
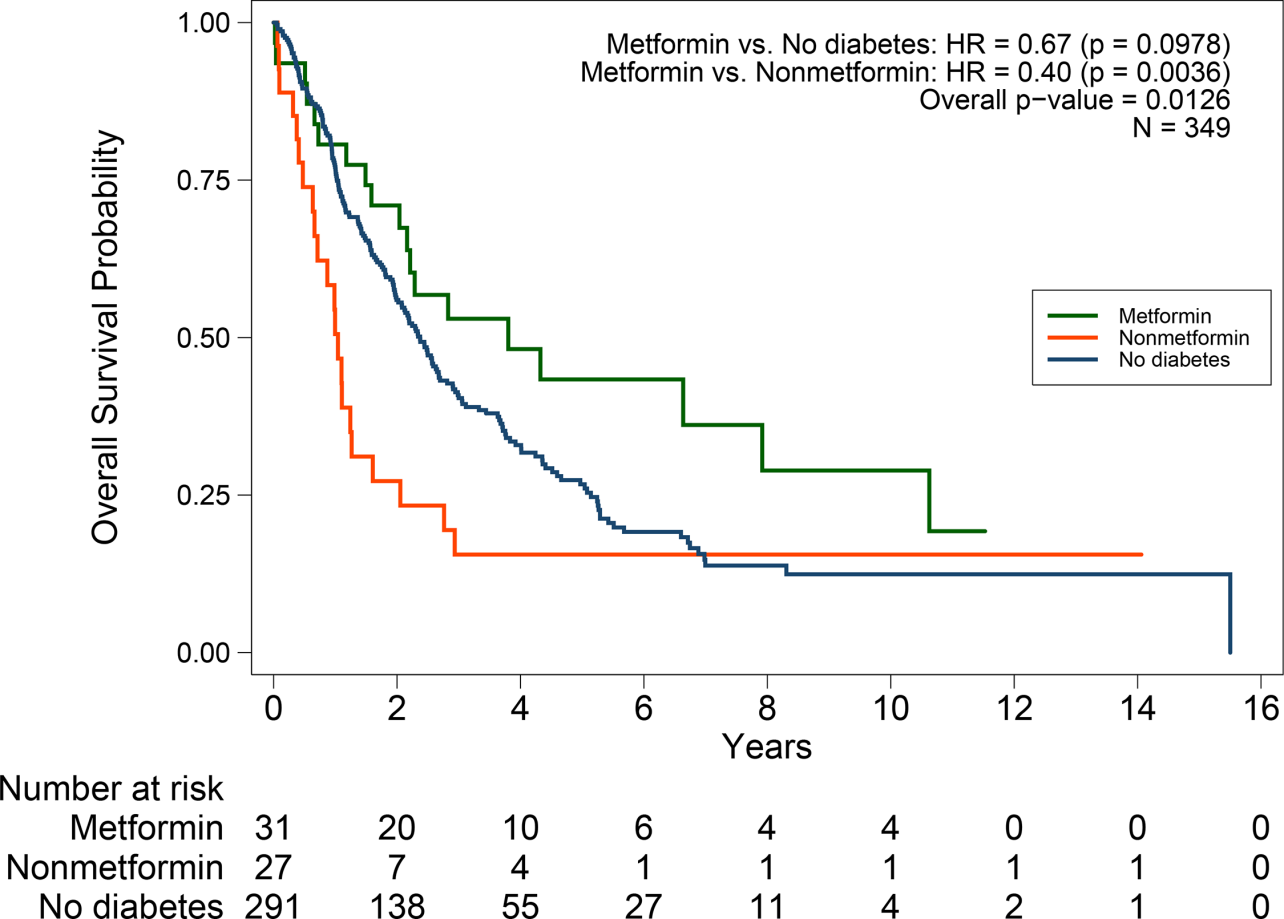
Kaplan-Meier estimates of overall survival. The three groups are: endometrial cancer patients with type II diabetes taking metformin; endometrial cancer patients without diabetes; and endometrial cancer patients with diabetes not taking metformin. *P* values are from the Cox model adjusting for study site.

Kaplan-Meier estimates of OS in the three groups and hazard ratio estimates based on a Cox regression model adjusted for study site are presented in Fig 2. OS was longest for patients with diabetes who used metformin. Median OS was 45.6 months (95%CI, 24.5–95.0) for patients with diabetes who used metformin, 28.5 months (95% CI, 23.9–32.1) for patients without diabetes, and 12.5 months (95% CI, 7.6–15.2 months) for patients with diabetes who did not use metformin. OS was significantly different between the three groups (log-rank test comparing the three groups *P =* 0.006). There was no association between having diabetes (if diabetic treatment was not considered) and overall survival (S1 Fig). The median overall survival is 28.5 months for subjects without diabetes compared to 24.5 months for patients with diabetes (P = 0.91).

A univariate Cox model (Table 2) showed that patients with diabetes who used metformin had better OS than patients with diabetes who did not use metformin (HR, 0.38; *P = 0*.002). The survival difference between the patients with diabetes who used metformin and the subjects without diabetes was not statistically significant (HR, 0.68; *P = 0*.109). Estimates adjusted for study site were similar (Fig 2) and there was no interaction between study site and metformin treatment group.

**Table 2:** Cox proportional hazards model estimates for overall survival.

Group	Univariate			Multivariate^*^		
	HR	95%, CI	*P*-value	HR	95%, CI	*P*-value
Metformin vs. Non-metformin	0.38	0.21–0.70	.002	0.42	0.23–0.78	.006
Metformin vs. No Diabetes	0.67	0.42–1.09	.109	0.65	0.41–1.05	.077
No Diabetes vs. Non-metformin	0.55	0.36–0.87	.010	0.65	0.41–1.01	.054
Overall (3 groups)	-	-	.007	-	-	.023

*Multivariate model adjusting for study site, stage (III vs. IV/Recurrent), and age at chemotherapy.

### Multivariate Regression Models

A multivariate Cox proportional hazards model was used to evaluate differences in OS between the three groups, adjusting for other known prognostic factors that were significantly associated with OS in univariate models (S2 Table). The initial full model included main effects of diabetes/metformin status, study site (UCMC vs. NSUHS), race (White vs. Black and Other), stage (III vs. IV/recurrent disease), obesity indicator and age at the time of chemotherapy administered for stage III/IV or recurrent disease. However, race and obesity were not found to be significant (*P = 0*.158 and *P* = 0.177, respectively), and the final model was adjusted only for study site, stage and age at the time of chemotherapy. The overall difference between the three groups was statistically significant (*P = 0*.023). In the multivariate analysis comparing the two groups with diabetes, metformin users had increased overall survival compared to diabetic patients who did not use metformin (HR, 0.42; *P = 0*.006). Overall survival rates did not differ significantly between metformin users and patients without diabetes (HR, 0.65; *P = 0*.077).(Table 2). The non-metformin group had a higher percentage of serous histology. However, inclusion of histologic subtype in the model did not change the results.

### Sub-group Analysis

**Fig 3:**
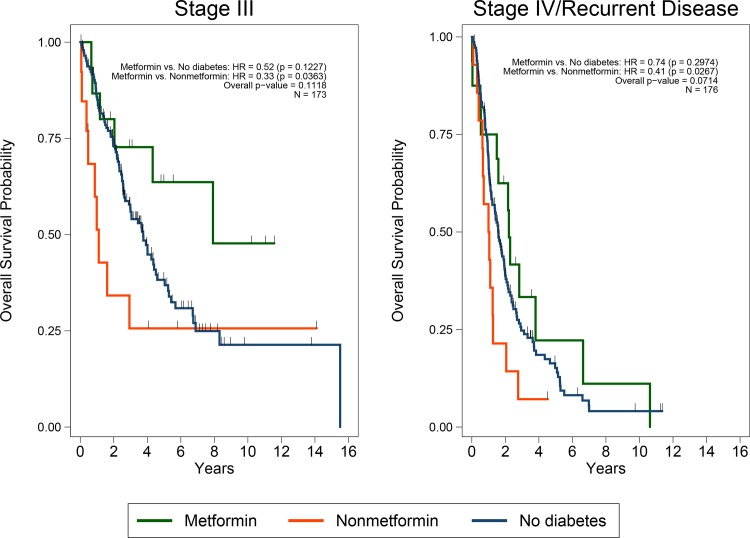
Kaplan-Meier estimates of overall survival. The three groups are: endometrial cancer patients with type II diabetes taking metformin; endometrial cancer patients without diabetes; and endometrial cancer patients with diabetes not taking metformin. *P* values are from the Cox model adjusting for study site. **(a)** Patients with stage III disease. **(b)** Patients with stage IV or recurrent disease.

To determine whether metformin’s association with improved survival was greater in stage III or stage IV/recurrent patients, a sub-group analysis was performed. We found that patients with diabetes who used metformin had better OS than patients with diabetes who did not use metformin both in the stage III (HR, 0.28; *P = 0*.017) and stage IV/recurrent (HR, 0.41; *P = 0*.028) sub-groups (Fig 3, estimates adjusted for site). In addition, the interaction between the groups and stage was not statistically significant in the multivariate Cox model adjusted for age and site (*P = 0*.69). However, this analysis is limited by the small sample size.

Initiating chemotherapy more quickly after diagnosis could improve chemotherapy response. To understand if metformin users received chemotherapy more quickly, a subgroup analysis was performed of patients with stage III/IV endometrial cancer (n = 306) and the amount of time that elapsed from the time of diagnosis to the start chemotherapy was calculated. The median number of days to starting chemotherapy was shortest for patients without diabetes (51 days), compared to 73 days for non-metformin users and 78 days for metformin users. The shorter interval to starting chemotherapy in the non-diabetic group compared to the patients with diabetes was statistically significant (P = 0.03), however, the interval to starting chemotherapy was similar between metformin users and non-metformin users (P = 0.58). This suggests that improved survival among metformin users is not a result of starting chemotherapy sooner; in fact, metformin users had longer survival despite having a prolonged interval the chemotherapy initiation.

## Discussion

The study presented here suggests a survival benefit for patients with stage III-IV or recurrent endometrial cancer who received chemotherapy while concurrently using metformin as a treatment for diabetes. In this retrospective cohort comparing patients with diabetes who used metformin, patients with diabetes who did not use metformin and patients without diabetes, patients using metformin had the longest survival (log-rank test comparing the three groups *P = 0*.006)(Fig 2). After adjusting for confounders, the patients who used metformin had a 58% lower hazard of death than patients with diabetes who did not use metformin. Overall, these findings in endometrial cancer add to the increasing preclinical evidence that metformin may have protective effects in multiple cancers.

The mechanism by which metformin might improve cancer survival remains to be elucidated. The patient cohort tested here was limited to patients treated with chemotherapy because it has been hypothesized that metformin acts as a chemosensitizer. The metformin users had improved survival despite having a prolonged interval to chemotherapy initiation. Preclinical testing in breast, prostate and lung cancer indicates that metformin improves response to several types of chemotherapy, allowing for a significant reduction in chemotherapy dose [25]. In breast cancer, a retrospective analysis of patients receiving neoadjuvant chemotherapy found that patients who were using metformin for diabetes had a 24% pathological complete response rate compared to 8% for patients with diabetes who were not using metformin and 16% for nondiabetics (*P = 0*.02) [26].

Independent of chemotherapy, metformin’s anticancer properties could be explained by a reduction in systemic insulin and glucose levels. There is evidence linking elevated insulin levels to tumorigenesis [27]. In this cohort, we did not have sufficient data on glycosylated hemoglobin A1C or glucose levels and, therefore, could not ascertain glucose control in the patients with diabetes. Others have attempted to clarify metformin’s systemic and tumor-direct effects in endometrial cancer using pre-operative window of opportunity trial designs [28–31]. In these trials, nondiabetic patients were prospectively treated with metformin (850–2250 mg/day) for 1 to 6 weeks prior to undergoing hysterectomy. Endometrial tissue samples and, in three studies, serum were collected at baseline and after metformin treatment. In two studies metformin treatment reduced serum insulin levels [30,31] and in one study it reduced serum glucose levels [31]. All of the studies, except one [29], reported that metformin had a direct effect on tumors by reducing proliferation of tumor cells (measured as levels of Ki-67 expression). These innovative study designs provide important insight into metformin’s potential mechanism of action in endometrial cancer.

Given the retrospective nature of our study, rigorous testing of metformin’s molecular mechanism of action is not possible. It is also important to note that all of the studies presented to date, including ours, have used death from all causes, not endometrial cancer-specific death, as the primary outcome. Therefore, one cannot definitively conclude that patients who use metformin are less likely to die from endometrial cancer, since it is possible that metformin decreases death from other causes (e.g. complications of diabetes and cardiovascular disease). However, in this cohort the baseline comorbidities in the two diabetic groups were similar, indicating that the metformin users did not have better overall health than non-metformin users. In addition, due to the aggressive nature of the disease, patients with stage III-IV or recurrent endometrial cancer are most likely to die from cancer.

An additional potential limitation of the study is that the designation of diabetes was made from retrospective chart review and not prospective testing and, therefore, the nondiabetic group could include undiagnosed diabetes. However, in this cohort, the diagnosis of diabetes (without consideration of diabetes treatment) was not associated with overall survival (S1 Fig). As summarized in a recent meta-analysis, the evidence supporting an association between diabetes and endometrial cancer mortality is inconclusive [32]. Lack of information regarding the duration of metformin use for the 6 metformin users that changed diabetes treatment during the follow-up period also restrains our study. Unique advantages of the study are that the patients in this cohort were treated at two different major medical centers and that 28% of the subjects were Black. These features increase the generalizability of our findings.

In conclusion, in this retrospective cohort study patients with diabetes who used metformin while being treated with chemotherapy for stage III-IV or recurrent endometrial cancer had improved overall survival. These findings add to the pre-clinical and epidemiologic data indicating that metformin may have a role in therapy for endometrial cancer. In fact, several clinical trials are underway evaluating metformin for endometrial cancer treatment, including a phase II/III trial in which patients with stage III-IV or recurrent endometrial cancer are being randomly assigned to treatment with metformin or placebo in addition to paclitaxel and carboplatin (NCT0265687) [33]. Given the results of retrospective studies, such as ours, these prospective randomized trials hold a great deal of promise. Ultimately, it is possible that metformin will be repurposed as an economical and well-tolerated adjuvant to chemotherapy for treatment of advanced endometrial cancer.

## Supporting Information

S1 Fig Kaplan-Meier estimates of overall survival. The two groups are: endometrial cancer patients with type II diabetes and endometrial cancer patients without diabetes.
(TIF)

S1 Table Comorbidities at baseline among patients with diabetes. (DOCX)

S2 Table Univariate cox model estimates for overall survival. (DOCX)
