# Supplementary figures and images for: Association of Metformin Use with Outcomes in Advanced Endometrial Cancer Treated with Chemotherapy
Source: PLoS One. 2016 Jan 20;11(1):e0147145. doi: 10.1371/journal.pone.0147145 (PMC4720394; doi:10.1371/journal.pone.0147145)

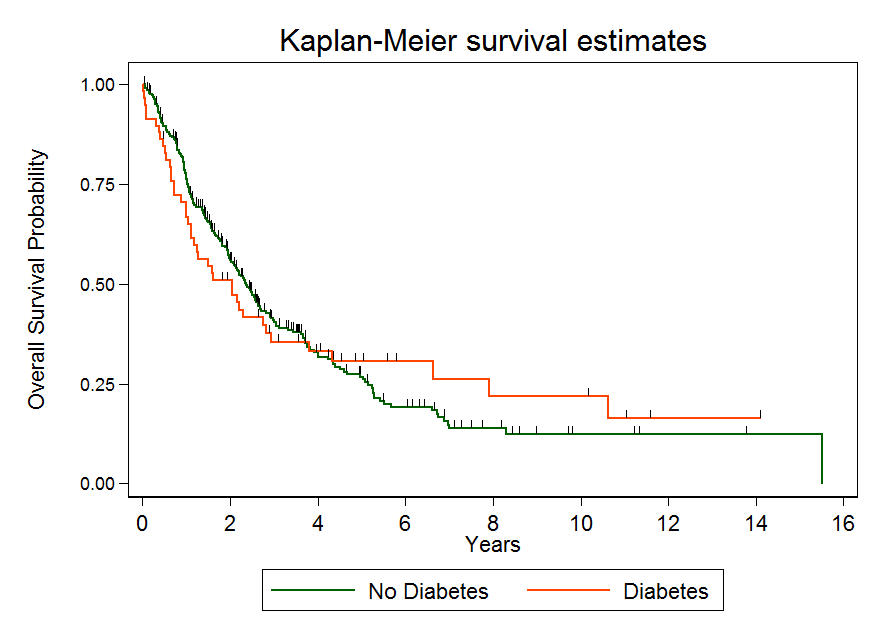

Supplement: S1 Fig — The two groups are: endometrial cancer patients with type II diabetes and endometrial cancer patients without diabetes. (TIF) [file pone.0147145.s001.tif]
