# Supplementary material for: Association of Metformin Use with Outcomes in Advanced Endometrial Cancer Treated with Chemotherapy
Source: PLoS One. 2016 Jan 20;11(1):e0147145. doi: 10.1371/journal.pone.0147145 (PMC4720394; doi:10.1371/journal.pone.0147145)
Supplement: S1 Table — (DOCX) [file pone.0147145.s002.docx]

**S1 Table.** Comorbidities at baseline among patients with diabetes.

|  | **Metformin Group**  **(N=31)** | **Nonmetformin Group**  **(N=27)** | **Total**  **(N=58)** | **P-value** |
| --- | --- | --- | --- | --- |
| **HTN** | 22 (71.0) | 24 (88.9) | 46 (79.3) | 0.115 |
| **HLD** | 10 (32.3) | 6 (22.2) | 16 (28.6) | 0.557 |
| **CVD** | 10 (32.3) | 5 (18.5) | 15 (25.9) | 0.368 |
| **Pulmonary** | 5 (16.1) | 4 (14.8) | 9 (15.5) | 1.0 |
| **Other Cancer** | 5 (16.1) | 5 (18.5) | 10 (17.2) | 1.0 |
| **Other** | 4 (12.9) | 4 (14.8) | 8 (13.8) | 1.0 |

CVD: CAD, CHF, MI, DVT, arrhythmia, and atrial septum aneurysm

Pulmonary: asthma, COPD, OSA, PE, sarcoidosis, and pulmonary hypertension

Other cancer: Hodgkin's lymphoma, breast and colon cancer

Other: CKD, depression, lupus, RA, hypothyroid, sickle cell
