# Supplementary material for: Association of Metformin Use with Outcomes in Advanced Endometrial Cancer Treated with Chemotherapy
Source: PLoS One. 2016 Jan 20;11(1):e0147145. doi: 10.1371/journal.pone.0147145 (PMC4720394; doi:10.1371/journal.pone.0147145)
Supplement: S2 Table — (DOCX) [file pone.0147145.s003.docx]

### S2Table. Univariate cox model estimates for overall survival.

|  |  | **HR** | **95% CI** | ***P*-value** |
| --- | --- | --- | --- | --- |
| **Site** |  |  |  |  |
|  | UCMC vs. NSUHS | 1.36 | 1.02-1.80 | .034 |
| **Race** |  |  |  |  |
|  | White Vs. Black/Other | 0.65 | 0.50-0.85 | .002 |
| **Stage** |  |  |  |  |
|  | IV/Recurrent vs. III | 2.34 | 1.80-3.04 | <.001 |
| **Age at Chemotherapy** |  | 1.03 | 1.02-1.05 | <.001 |
| **BMI** |  |  |  |  |
|  |  | 1.00 | 0.98-1.02 | 0.898 |
| **Obese** |  |  |  |  |
|  | BMI>30 vs BMI<30 | 1.11 | 0.85-1.46 | 0.448 |
| **Smoking History (n=272)** |  |  |  | .454 |
|  | Current Smoker vs. Never-Smoker | 0.95 | 0.57-1.60 | .851 |
|  | Former Smoking vs. Never-Smoker | 1.21 | 0.88-1.66 | .246 |
